# Supplementary material for: Efficient and Tidy Manipulation of Annotated Matrix Data with plyxp
Source: bioRxiv. 2026 May 11:2026.05.06.721669. Preprint. [Version 1] doi: 10.64898/2026.05.06.721669 (PMC13192902; doi:10.64898/2026.05.06.721669)
Supplement: Supplement 2 [file NIHPP2026.05.06.721669v1-supplement-2.pdf]

bioRxiv preprint doi: https://doi.org/10.64898/2026.05.06.721669; this version posted May 11, 2026. The copyright holder for this preprint (which was not certified by peer review) is the author/funder, who has granted bioRxiv a license to display the preprint in perpetuity. It is made available under a CC-BY 4.0 International license.

Supplementary Note

Interoperability with *tidySummarizedExperiment*

plyxp enables users to continue using the *tidySummarizedExperiment* package for operating directly on SE objects using *dplyr* verbs without namespace conflicts, as it employs a class *PlySummarizedExperiment* to trigger its methods. In practice, the user instantiates a *PlySummarizedExperiment* object by providing an SE to the `new_plyxp()` function. The *PlySummarizedExperiment* class is related to the SE class by composition in that *PlySummarizedExperiment* contains a single slot which accepts any object inheriting from the *SummarizedExperiment* class. Due to composition, *plyxp* can generalize its core functionalities to more specialized sub classes of the *SummarizedExperiment* class, such as *SingleCellExperiment* class. Meanwhile, *tidySummarizedExperiment* users can continue to use *dplyr* verbs as previously without any change in behavior.

Performance Considerations

Abstracting a user's entry point into annotated matrix objects provides several advantages. To emphasize these advantages, we will first consider a counterexample. We will require an isomorphic implementation, meaning that both input and output are the same object classes. A sensible approach is to unwind the annotated matrix into a tabular structure and then reconstruct the original annotated matrix before returning. An advantage of this framework is that it allows the use of standard tabular processing libraries like *dplyr* or *pandas* directly. However, it has notable drawbacks that depend on the complexity of the user's object.

Under this unwinding model, one can expect a large amount of overhead dedicated to transforming the object into the tabular structure required for a tabular processing library. In the worst case scenario, this transformation is done eagerly for all data in the object per method, which would be a significant amount of work done before any user expressions are evaluated. Another consideration for constructing this tabular structure would be the possibility of symbol collision, that is, how to resolve the resulting column names if the input contexts have identical names. In order to work, some heuristic would need to be established such as a renaming convention or context precedence. In either case in which these collisions would occur, the resulting code would be ambiguous, and it may be better to send an error and enforce uniqueness across the entire object than force the user to handle heuristic name resolution. The final issue with the conversion is the loss of information regarding where data is from and where it should be set. In the case of *dplyr* or *pandas* verbs that modify the object, `mutate()` or `.assign()` respectively, it is unclear where new data should be set in the reconstructed annotated matrix object. A safe choice would be to set the new data as a new assay, however, if the implementation would need to set the result in either the row or column metadata tables, then additional computation would be required to ensure the resulting new vector's uniqueness and its ability to fit in one of those positions.

In *plyxp*, all issues related to ambiguity are resolved by constructing three separate data masks. *plyxp* imposes a verbose, but specific syntax to communicate intent of which context an expression should be evaluated. Under this model, there is no additional computational cost for direct access, no possibility of symbol collision, and no ambiguity about where data will be set on the output object. Only symbols bound within the contexts the user requests are evaluated, saving time compared to approaches that manipulate the entire structure all together. If the user requires cross-context data in which the requested data may be transformed, these bindings are also lazy and save computation time if never forced. This is especially convenient for grouping operations, in which constructing groups eagerly for a dense object would require extensive computation. In this model's design of the data masks, the cost of performing this computation is only paid once per data mask per symbol.

Finally, *plyxp* makes some assumptions about how the user may want their data to be shaped in a given context, but provides the user with a way to access the underlying data without reshaping. This is motivated by the fact that, despite utilizing efficient subset functions when possible, it may still be more efficient for the user to operate on the native data structure with optimized routines, opting to forego any potentially expensive transformations.
